# Impaired retinal oxygen metabolism and perfusion are accompanied by plasma protein and lipid alterations in recovered COVID-19 patients

**DOI:** 10.1038/s41598-024-56834-4

**Published:** 2024-04-10

**Authors:** Viktoria Pai, Andrea Bileck, Nikolaus Hommer, Patrick Janku, Theresa Lindner, Victoria Kauer, Benedikt Rumpf, Helmuth Haslacher, Gerhard Hagn, Samuel M. Meier-Menches, Leopold Schmetterer, Doreen Schmidl, Christopher Gerner, Gerhard Garhöfer

**Affiliations:** 1 Department of Clinical Pharmacology, Medical University of Vienna, Währinger Gürtel 18-20, 1090 Vienna, Austria; 2 Department of Analytical Chemistry, Faculty of Chemistry, University of Vienna, Währinger Straße 38, 1090 Vienna, Austria; 3 Joint Metabolome Facility, University of Vienna and Medical University Vienna, Vienna, Austria; 4 Department of Medicine IV for Infectious Diseases and Tropical Medicine, Clinic Favoriten, Vienna, Austria; 5 Department of Laboratory Medicine, Medical University of Vienna, Vienna, Austria; 6 Institute of Inorganic Chemistry, Faculty of Chemistry, University of Vienna, Vienna, Austria; 7 Singapore Eye Research Institute, Singapore National Eye Centre, Singapore, Singapore; 8 Ophthalmology and Visual Sciences Academic Clinical Program, Duke-NUS Medical School, Singapore, Singapore; 9 SERI-NTU Advanced Ocular Engineering (STANCE), Singapore, Singapore; 10 School of Chemistry, Chemical Engineering and Biotechnology, Nanyang Technological University, Singapore, Singapore; 11 Center for Medical Physics and Biomedical Engineering, Medical University of Vienna, Vienna, Austria; 12 Institute of Molecular and Clinical Ophthalmology, Basel, Switzerland

**Keywords:** SARS-CoV2, COVID-19, Ocular blood flow, Metabolomics, Multi-omics analysis, Biomarkers, Viral infection

## Abstract

The aim of the present study was to investigate retinal microcirculatory and functional metabolic changes in patients after they had recovered from a moderate to severe acute COVID-19 infection. Retinal perfusion was quantified using laser speckle flowgraphy. Oxygen saturation and retinal calibers were assessed with a dynamic vessel analyzer. Arterio-venous ratio (AVR) was calculated based on retinal vessel diameter data. Blood plasma samples underwent mass spectrometry-based multi-omics profiling, including proteomics, metabolomics and eicosadomics. A total of 40 subjects were included in the present study, of which 29 had recovered from moderate to severe COVID-19 within 2 to 23 weeks before inclusion and 11 had never had COVID-19, as confirmed by antibody testing. Perfusion in retinal vessels was significantly lower in patients (60.6 ± 16.0 a.u.) than in control subjects (76.2 ± 12.1 a.u., *p* = 0.006). Arterio-venous (AV) difference in oxygen saturation and AVR was significantly lower in patients compared to healthy controls (*p* = 0.021 for AVR and *p* = 0.023 for AV difference in oxygen saturation). Molecular profiles demonstrated down-regulation of cell adhesion molecules, NOTCH3 and fatty acids, and suggested a bisphasic dysregulation of nitric oxide synthesis after COVID-19 infection. The results of this study imply that retinal perfusion and oxygen metabolism is still significantly altered in patients well beyond the acute phase of COVID-19. This is also reflected in the molecular profiling analysis of blood plasma, indicating a down-regulation of nitric oxide-related endothelial and immunological cell functions.

*Trial Registration*: ClinicalTrials.gov (https://clinicaltrials.gov) NCT05650905.

## Introduction

The severe acute respiratory syndrome coronavirus 2 (SARS-CoV-2) pandemic starting in the year 2019 has led to an unprecedented worldwide health crisis, which is still ongoing. Although the introduction of SARS-CoV-2 vaccines has decreased the mortality of an acute infection^1^, the SARS-CoV-2 pandemic is still a considerable health burden for all countries worldwide. It has become clear that beside the acute SARS-CoV-2 infection, which mainly affects the respiratory system, the post-acute sequelae of an infection may result in persisting symptoms for several months, which is usually referred to as long-coronavirus disease 2019 (COVID-19) syndrome^2^.

Strikingly, recent data from large cross-sectional studies indicates that even individuals that have fully recovered from a past COVID-19 infection without any remaining symptoms are still at considerably increased risk of incident cardiovascular disease, cerebrovascular disorders, thromboembolic events and other vascular related complications^3–5^. This also holds true for asymptomatic individuals or patients who were not hospitalized during the acute phase of the infection and are therefore considered as mild to moderate cases^3^.

The exact reason for the increased cardiovascular event-rate in the post-acute phase after COVID-19 infection is still unclear. However, there is mounting evidence now that the SARS-CoV-2 virus causes endothelial dysfunction in the pan-vascular endothelium, either by a direct infection of the SARS-CoV-2 virus of endothelial cells or by the well described cytokine storm during the COVID-19 infection^6^. As the vascular endothelium is the main interface between the blood stream and the surrounding tissue, a virus-induced endothelial dysfunction would have detrimental consequences including impaired regulation of vascular tone, vascular hyperpermeability, hypercoagulability, thrombosis and others. Thus, it has been hypothesized that in particular the post-acute consequences of a COVID-19 infection may be related to microvascular impairment and endothelial dysfunction^6,7^.

As the human eye offers the unique possibility to visualize the microvasculature, the current study seeks to test the hypothesis that microvascular functional changes can be detected even after recovery from COVID-19. For this purpose, ocular perfusion parameters and retinal oxygen metabolism were assessed non-invasively in patients who had recovered from moderate to severe COVID-19 infection within the last 6 months without sequelae before inclusion in this study and compared to a group of healthy age- and sex-matched controls. Furthermore, multi-omics analysis of blood plasma was carried out as these methods are ideal to monitor systemic effects of inflammation^8^. Only recently, we have presented a molecular signature characteristic for long COVID-19 syndrome using these postgenomic analysis methods^9^. Here, we are applying these methods to obtain evidence for persisting systemic imbalance in recovered COVID-19 patients that would support the observations in the local ocular microvasculature.

## Methods

### Participants

The study protocol was approved by the Ethics Committee of the Medical University of Vienna (EC No. 1647/2020) on 03.07.2020 and was conducted in compliance with the Declaration of Helsinki and the Good Clinical Practice (GCP) guidelines of the European Union. The recruitment period lasted from July 2021 to March 2022. All study participants provided written informed consent and all measurements were performed in patients as well as in healthy age- and sex-matched controls. Patients were selected by the Department of Medicine IV at the Klinik Favoriten while healthy control subjects were recruited by the Department of Clinical Pharmacology at the Medical University of Vienna. Patients had to have a history of moderate to severe COVID-19 according to the World Health Organization (WHO) criteria^10^ and no symptoms or diagnosis of long COVID-19 syndrome. All included subjects passed a screening examination in the two weeks before the study day consisting of medical history including symptoms and severity of COVID-19 infection in patients, height, weight, blood pressure and heart rate measurement. In addition, a blood draw for evaluation of SARS-CoV-2 seroprevalence using nucleocapsid antibody tests, spike protein IgG antibody test, plasma metabolomics and lipidomics was performed. In order to exclude acute SARS-CoV-2 infections, polymerase chain reaction (PCR) testing was carried out. Further, all participants underwent an ophthalmic examination including assessment of visual acuity, slit lamp biomicroscopy, indirect funduscopy and measurement of intraocular pressure (IOP) with Goldmann applanation tonometry. Subjects were excluded if any clinically significant abnormality preventing reliable ocular measurements was found, such as for example severe cataract, diabetic retinopathy, glaucoma, age-related macular degeneration or amblyopia, or if they had donated blood in the 3 weeks before inclusion. The eye with the better visual acuity was chosen as study eye and if there was no difference between eyes in terms of visual acuity, the right eye was used as study eye. Patients or control subjects with systemic diseases such as arterial hypertension or diabetes were only included if those were stable and well-controlled.

### Study design

The study was conducted in an observer-masked design, meaning the outcomes assessor was not aware of the diagnosis of the analyzed subject. At the beginning of the study day a pregnancy test was performed in females of childbearing potential and one drop of 0.5% tropicamide (Mydriaticum “Agepha”, Agepha, Vienna, Austria) was instilled in the study eye. After a resting period of at least 20 min to ensure stable hemodynamic conditions and sufficient mydriasis, retinal nerve fiber layer thickness (RNFLT) and vessel density were measured using a commercially available spectral domain optical coherence tomography (OCT) system with an OCT-Angiography (OCT-A) module (Heidelberg Spectralis OCT, Heidelberg Engineering, Heidelberg, Germany). Then, retinal vessel diameters and oxygen saturation was assessed with the dynamic vessel analyzer (DVA). Ocular blood flow was measured using laser speckle flowgraphy (LSFG). Blood pressure, heart rate and IOP were measured immediately before and after all ocular blood flow measurements had been performed.

### Outcomes assessments

#### Noninvasive measurement of systemic hemodynamics

Systolic, diastolic and mean arterial pressure were measured at the upper arm by an automated oscillometric device (Infinity Delta; Dräger, Vienna, Austria). The same device recorded pulse rate and peripheral oxygen saturation using a fingertip pulse oximeter.

#### Intraocular pressure

A slit-lamp mounted Goldmann applanation tonometer was used to measure IOP. One drop of oxybuprocainhydrochloride combined with sodium fluorescein was used for anesthesia of the cornea before each measurement.

#### Evaluation of SARS-CoV-2-seroprevalence

To differentiate between COVID-19 recovered and vaccinated participants SARS-CoV-2 seroprevalence was evaluated using nucleocapsid antibody tests and spike protein IgG antibody tests^11^.

#### PCR testing for acute SARS-CoV-2 infection

Nasopharyngeal swabs were obtained according to the instructions of the Center for Virology, Medical University of Vienna. PCR testing was performed at the Center for Virology, Medical University of Vienna.

#### Plasma profiling

In order to investigate whether systemic molecular alterations may be associated with the altered vascular properties consequent to COVID-19 infection, plasma samples underwent a multi-omics analysis based on mass spectrometry as described previously^9^. In short, proteome profiling was accomplished using a Dionex Ultimate 3000 nano high performance liquid chromatography (HPLC)-system (Thermo Fisher Scientific) hyphenated with a timsTOF Pro mass spectrometer (Bruker). Fatty acids and their oxidation products (eicosadomics) were analysed using a Thermo ScientificTM VanquishTM (UHPLC) system hyphenated with a Q ExactiveTM HF Quadrupole-OrbitrapTM high-resolution mass spectrometer (Thermo Fisher Scientific). Peak areas of eicosanoids and fatty acids were normalized to the internal standards. Afterwards, 20 was added to the log2-transformed normalized peak areas via the MinProb function of the imputeLCMD package (version 2.1)^12^, resulting in adjusted normalized areas under the curve (ad_nAUC) values.

The metabolome analyses were performed using a ExionLC AD chromatography system (AB Sciex, Framingham, MA, USA) hyphenated with a Sciex 6500 + series QTRAP mass spectrometer and the commercial MxP Quant 500 kit (Biocrates, Innsbruck, Austria) covering up to 630 metabolites from 26 biochemical classes. The kit was processed according to the protocol provided by the manufacturer.

#### Optical coherence tomography and OCT-A

RNFLT was assessed using a commercially available spectral domain OCT system (SD-OCT, Heidelberg Spectralis OCT, Heidelberg Engineering, Heidelberg, Germany). Scans were performed using the glaucoma ONH-RC scanning protocol (15° peripapillary field with a size of 768 × 496 pixels).

For OCT-A images, macula-centered high-resolution (512 B-scans, 512 A-scans/B-scan) 10° × 10° scans were carried out. The layers of interest used in the study were superficial vascular plexus (SVP), intermediate vascular plexus (ICP), and deep capillary plexus (DCP). The scans were subject to a quality check to filter out images of insufficient quality. The raw scans were then analyzed using Matlab version 2023b software (MathWorks, Natick, MA), where the images were binarized using the mean value and in the SVP the large Vessels (LV) where marked and then the perfusion densities of capillaries and LV were calculated.

#### Dynamic vessel analyzer

For measurement of retinal vessel calibers, a commercially available DVA ( IMEDOS, Jena, Germany) was used which comprises of a fundus camera, a video camera, a real time monitor and a personal computer with an analyzing software for the accurate determination of retinal arterial and venous diameters^13^. Based on these diameter measurements, central retinal arterial equivalent (CRAE), central retinal venous equivalent (CRVE) and arteriovenous ratio (AVR) according to the Parr‐Hubbard formula was calculated^14,15^.

Measurement of retinal oxygen saturation was performed with the same device using the image analysis software of the DVA camera (Visualis 3.0). Briefly, two monochromatic fundus images, obtained by the camera and a filter assembly, were taken at different wavelengths of 610 nm and 540 nm simultaneously. Since hemoglobin exerts different light absorption characteristics depending on its level of oxygenation, oxygen saturation in retinal arteries and veins can be estimated by using the difference between these two images. The operator marked all vessels surrounding the optic nerve head and the software automatically provided mean oxygen saturation values for all marked veins and arteries. Arteriovenous difference in oxygen saturation was then simply calculated in subtracting venous oxygen saturation from arterial oxygen saturation^16,17^.

#### Laser speckle flowgraphy

For measurement of optic nerve head blood flow, a commercially available LSFG (Nidek, Japan) was used. The software of the system (LSFG Analyzer, Version 1.0.1.1) provides the values mean area flow rate (MA), mean tissue flow rate (MT) and mean vessel flow rate (MV). MT refers to blood flow in the tissue of the optic nerve head while MV refers to the main vessel area. MA is the composite of MT and MV^18,19^.

### Statistical analysis

Statistical analysis was performed using IBM SPSS Statistics (Version 26, IBM, Armonk, New York, USA). All values are presented as means ± SD. Normal distribution for all major outcome variables was confirmed using the Kolmogorov–Smirnov test. Descriptive statistics are reported for all values obtained. A chi-square test was used to compare frequencies of concomitant diseases between groups. The Levene test to assess the equality of variances was performed for all outcome variables*.* Then, unpaired t-tests between groups were calculated and depending on the results of the Levene test, pooled or unpooled variances and a correction to the degrees of freedom were used. A *p* value < 0.05 was considered as the level of significance.

The publicly available Perseus software (version 1.6.14.0) was used for the statistical analysis of proteins as well as metabolites^20^. For each data set, intensity values of identified molecules were log2 transformed and missing values imputed by normal distribution. Thereafter, principal component analysis (PCA) as well as unpaired, multi-parameter corrected, t-tests between the study groups applying an FDR of 0.05 and a S0 of 0.1 was performed separately for each omics data set.

## Results

A total of 40 subjects were included in the present study, of which 29 had recovered from moderate to severe COVID-19 within 2 to 23 weeks before inclusion. Eleven control subjects never had COVID-19, as confirmed by antibody testing. Mean age was comparable with 35 ± 16 years in the COVID-19 group and 36 ± 12 years in the control group (*p* = 0.777). In the COVID-19 group, 13 subjects were female and 16 subjects were male and in the control group 6 subjects were female and 5 subjects were male. No difference between groups was found in terms of concomitant diseases, such as arterial hypertension (p = 0.288), asthma bronchiale (*p* = 0.814) or diabetes mellitus (*p* = 0.267). A table showing all concomitant diseases of the COVID-19 group that were present at screening is provided in the supplement (S-Table 1). Systolic, diastolic and mean arterial blood pressure was within normal ranges and similar for the two groups (*p* > 0.1 each between groups). Body mass index (BMI) was significantly higher in the COVID-19 group (27.5 ± 5.6 kg/m^2^ vs. 24.5 ± 2.8 kg/m^2^, *p* = 0.036). No difference in RNFLT was found between groups (102.9 ± 8.8 µm in the COVID-19 group and 103.7 ± 9.0 µm in the control group, *p* = 0.784).

### Retinal vascular parameters

**Figure 1:**
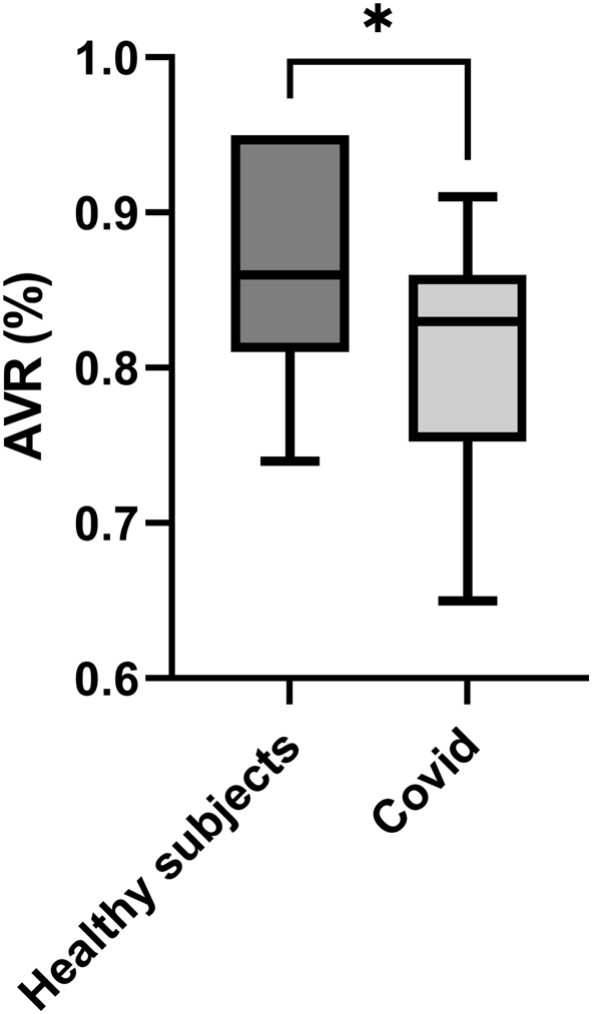
Arteriovenous ratio in vessel diameter in healthy subjects and patients that had recovered from COVID-19. The top of the box represents the 75th percentile, the bottom of the box represents the 25th percentile, and the line in the middle represents the 50th percentile. The whiskers represent the highest and lowest values. *p < 0.05 between groups.

**Figure 2:**
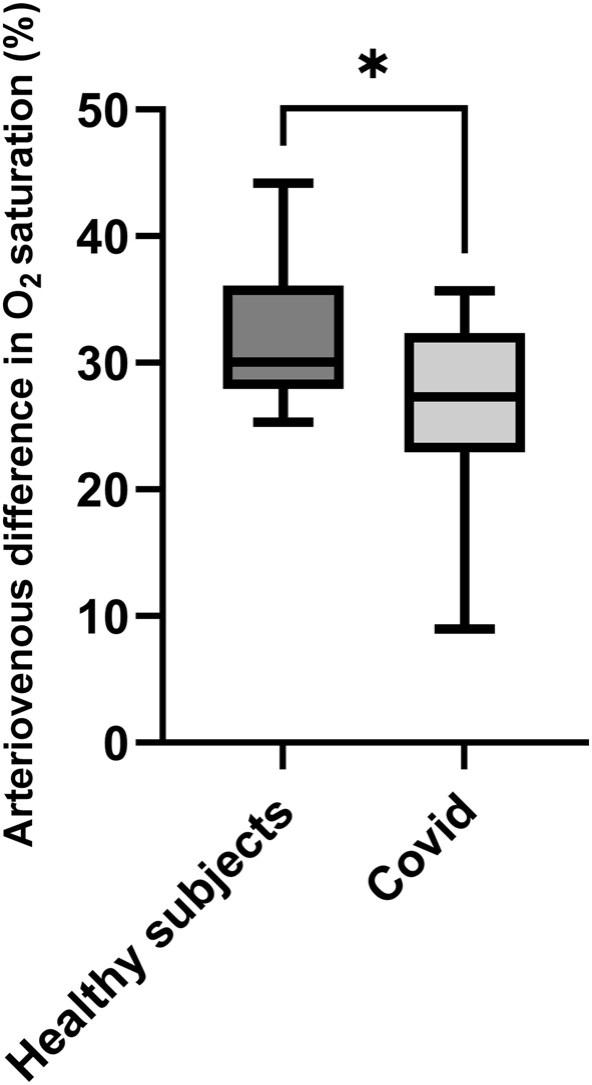
Arteriovenous difference in oxygen saturation in healthy subjects and patients that had recovered from COVID-19. The top of the box represents the 75th percentile, the bottom of the box represents the 25th percentile, and the line in the middle represents the 50th percentile. The whiskers represent the highest and lowest values. *p < 0.05 between groups.

While no significant difference between CRAE (184.6 ± 14.2 µm in the COVID-19 group vs. 189.4 ± 14.0 µm in healthy controls, *p* = 0.355) and CRVE (226.1 ± 22.3 µm vs. 217.5 ± 13.5 µm, *p* = 0.236) was found between groups, AVR was significantly lower in the COVID-19 group (0.81 ± 0.06 vs. 0.87 ± 0.08, *p* = 0.021, Fig. 1). In addition, arteriovenous difference in oxygen saturation was significantly lower in patients that had recovered from COVID-19 (26.8 ± 6.5%) compared to healthy controls (32.0 ± 5.5%, *p* = 0.023, Fig. 2). In contrast, no difference in arterial or venous oxygen saturation was found (*p* = 0.429 for retinal arteries and *p* = 0.092 for retinal veins). In the COVID-19 group, retinal arterial oxygen saturation was 95.3 ± 3.3% and venous oxygen saturation was 68.5 ± 6.8% while in the control group, retinal arterial oxygen saturation was 96.2 ± 3.4% and venous oxygen saturation was 64.2 ± 7.5%.

**Figure 3:**
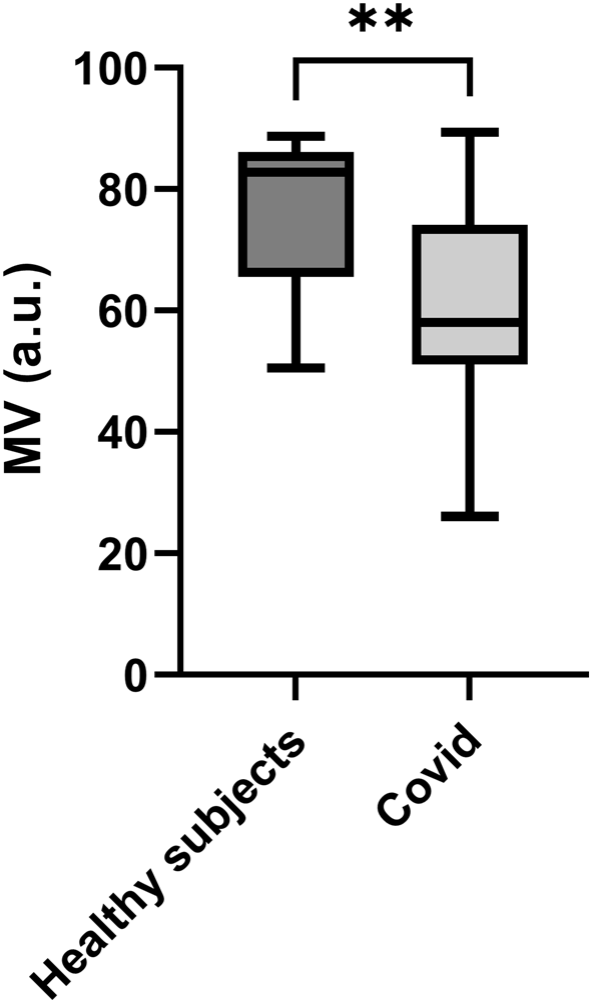
MV in retinal vessels in healthy subjects and patients that had recovered from COVID-19. The top of the box represents the 75th percentile, the bottom of the box represents the 25th percentile, and the line in the middle represents the 50th percentile. The whiskers represent the highest and lowest values. **p < 0.01 between groups.

MV was significantly lower in patients (60.6 ± 16.0 a.u.) compared to healthy controls (76.2 ± 12.1 a.u., *p* = 0.006, Fig. 3). In contrast, no statistically significant difference in MT (*p* = 0.109) or MA (*p* = 0.209) was found between groups, although both values tended to be lower in patients previously infected with COVID-19. MT was 23.0 ± 9.1 a.u. in the COVID-19 group and 28.1 ± 7.5 a.u. in healthy controls. MA was 36.5 ± 9.9 a.u. in the COVID-19 group and 40.7 ± 7.4 a.u. in the control group.

Large vessel density as measured by OCT-A was significantly lower in the COVID-19 group compared to healthy controls (7.3 ± 1.8% vs. 9.0 ± 2.2%, *p* = 0.028). In contrast, no difference in vessel density in retinal capillaries in the SVP, ICP or DCP was found (data not shown).

### Plasma fatty acids

**Figure 4:**
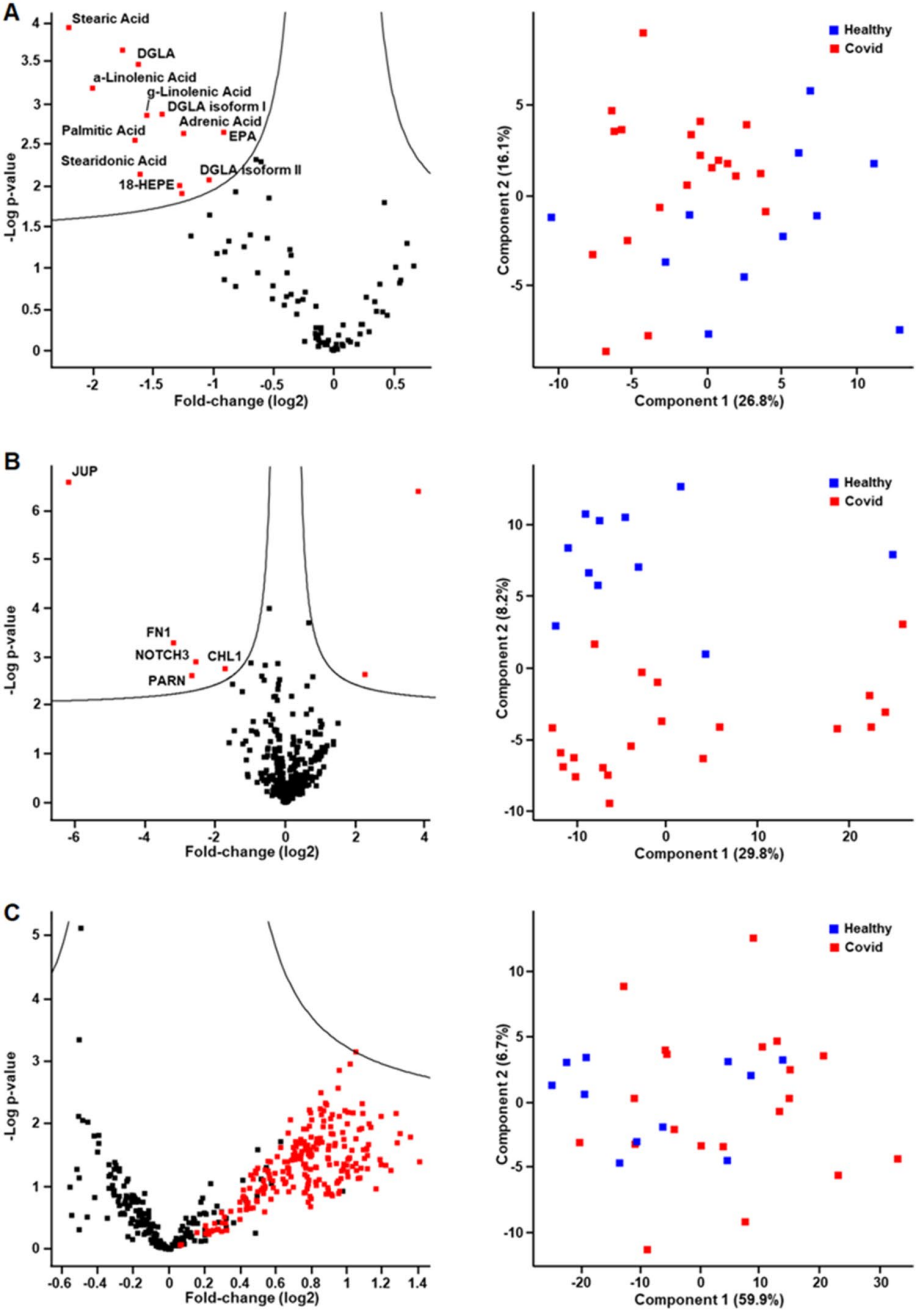
Systemic molecular alterations consequent to COVID-19 infection are visualized by volcano plots (left) and principal component analysis (PCA, right). Volcano plots of (**A**) eicosanoids and fatty acids as well as (**B**) proteins display significant changes of molecules in red. (**C**) Volcano plot of metabolites shows no significant changes but a uniform up-regulation of triglycerides marked in red.

The untargeted analysis of fatty acids and their oxidation products identified 88 molecules, 13 of which were found significantly deregulated (FDR < 0.05) (Fig. 4A, S-Table 2 and 3). A PCA again indicated almost complete group separation pointing to characteristic molecular alterations. Palmitic acid, stearic acid, stearidonic acid, dihomogammalinolenic acid (DGLA), alpha- and gamma linolenic acid, adrenic acid and eicosapentaenoic acid (EPA) as well as the anti-inflammatory CYP-product 18-HEPE were found down-regulated in the COVID-19 group.

### Plasma proteomics

Upon restricting to proteins identified in at least 5 individuals of a group with at least two distinct peptides, 379 proteins were identified and evaluated in plasma. Despite the well-known inter-individual variation of plasma proteins, a principle component analysis clearly separated the two groups, pointing to long-lasting molecular alterations potentially associated with the previous infection (Fig. 4B). While this method has a strong capacity to detect inflammatory events, no acute or chronic inflammation marker was found deregulated after complete recovery from COVID-19 infection. The only up-regulated proteins (FDR < 0.05) were antibodies, quite plausibly related to the previous infection. Amongst others, the cell adhesion molecules JUP (junction plakoglobin) and CHL1 (neural cell adhesion molecule L1-like protein), and the vascular smooth muscle cell protein NOTCH3 were found down-regulated.

### Metabolomics

A total of 462 metabolites were successfully quantified. While a PCA analysis indicated a rather poor group separation, and no single metabolite was found significantly altered, all 228 triglycerides covered by the analysis tended to higher concentration levels in recovered COVID-19 patients compared to healthy controls (Fig. 4C).

## Discussion

The present data indicates that patients who have clinically fully recovered from a SARS-CoV-19 infection still show altered retinal blood flow and oxygen metabolism up to 6 months after the acute infection. Thus, here we present evidence for a generalized vasoconstrictor state of the retinal microcirculation, which is accompanied by an altered retinal oxygen metabolism in the retinal tissue. Indeed, our data strongly supports the hypothesis that a long-term microcirculatory impairment is still present up to 6 months after COVID-19 infection.

Although during the acute infection, the clinical picture of COVID-19 is mainly driven by respiratory symptoms, ocular and systemic complications during and, in particular, after recovery from COVID-19 are of increasing concern^21–23^. Data from a variety of studies consistently show an increased risk of long-term cardiovascular events after a COVID-19 infection^3,24^. Recent data indicates that these events occur largely independent of the severity of symptoms during the SARS-CoV-2 infection. This seems to be closely related to the pathogenesis of the disease. The SARS-CoV-2 virus binds to the angiotensin-converting enzyme 2 (ACE2) receptor, which is a key entrance point of the virus to variety of cells including type 2 alveolar cells, bronchial epithelial cells but also endothelial cells and pericytes^25,26^. It has become clear that during a COVID-19 infection, endothelial damage and the resulting vascular dysfunction is a key pathogenetic factor during the acute infection^6,7,27^. Further, there is evidence that this holds also true for the post-acute complication of the disease: As vascular endothelium regulates vascular tone, platelet activity, the fibrinolytic system and other important factors of the vascular homoeostasis via the specific release of endothelium derived agents, the sustained functional impairment of the vascular endothelium after a COVID-19 may lead to the observed clinical manifestations even after clinical restitution^6,27^.

### Ocular perfusion

The retinal perfusion analysis results support the hypothesis of a microvascular impairment in COVID-19-patients after clinical recovery. In particular, the present data shows a shift in vascular tone which manifests in a significantly lower AVR of retinal vessels in post-COVID-19 patients when compared to healthy controls. Thus, it seems that the retinal microcirculation in post-COVID-19 patients is in a state of vasoconstriction when compared to patients who have not gone through a COVID-19 infection. This vasoconstrictor effect leading to a decreased AVR is well known from other systemic diseases that are associated with endothelial dysfunction such as arterial hypertension or coronary heart disease. Large cohort studies such as the Cardiovascular Health Study, the Beaver Dam Eye Study and others report that a reduced AVR is associated with an increased risk of developing cardio-vascular disease^28^ and that retinal vessel diameters are predictive for cardio-vascular risk in this group of patients^29^. Thus, one may hypothesize that changes in retinal vascular calibers could serve as biomarkers for systemic cardio-vascular risk in post-COVID-19 patients.

### Oxygen metabolism

Further, the results of the current study indicate a reduced arterio-venous difference in oxygen saturation in patients that had recovered from COVID-19 when compared to controls. This decrease of the arterio-venous difference may either be caused by a decreased oxygen consumption of the tissue or reduced oxygen uptake in the retina. As for the current study,microvascular impairment in post-COVID-19 patients may reduce the oxygen delivery to the tissue, which in turn would translate to a decreased arterio-venous difference in oxygen saturation. This hypothesis is also supported by previous studies using OCT-A that are consistently reporting a reduced microvascular density within in the retinal tissue^30,31^. Thus, it is reasonable to assume that a capillary dropout and capillary nonperfusion post COVID-19 infection may lead to a decrease of oxygen delivery to the tissue, which is then reflected in the decrease of oxygen extraction as found in the current study. Along this line of thought, a decrease in arterio-venous difference has also been found in other diseases associated with capillary non-perfusion such as diabetes^32^ or Alzheimer´s disease^33^. However, to finally prove this hypothesis, methods for the in vivo assessment of oxygen saturation on the level of retinal capillaries would be required, which currently do not exist.

However, a substantial decrease of oxygen delivery to tissue is also indicated by the findings regarding the blood flow measurements: Our results show a decreased mean retinal blood flow rate assessed with the LSFG technique in post-COVID-19 patients compared to subjects with no prior COVID-19 infection. In particular, our data shows reduced MV values, whereas MA and MT showed a tendency towards a reduction without reaching level of significance. Given that MV mainly represents the major vessels of the retinal circulation, whereas MA and MT may also contain signal from the deeper vascular layers, our data is well compatible with the hypothesis of a reduced retinal blood flow in post-COVID-19 patients. This is further supported by our findings using OCT-A, where a reduction in large vessel density was found, while there was no difference in vessel density in capillaries. This is in contrast to some other studies reporting prolonged disturbed microvasculature after COVID-19 infection measured with OCT-A^34–36^. However, this could be due to the inclusion of different study populations or other follow-up times than in the present study.

### Multi-omics profiling

Multi-omics profiling of blood plasma may provide relevant information regarding systemic alterations characteristic for the metabolic and vascular imbalances observed in recovered COVID-19 patients. As the function of the micro-vasculature is tightly regulated by proteins and metabolites, an analysis of such molecules was indicated. Principal component analyses visualized the variance of molecules identified in all samples: the more similar two samples, the closer the corresponding points in the PCA-plot (Fig. 4A,B). PCA clustered the two groups, demonstrating that the molecules causing the highest variation between samples separated the patient groups quite well. This observation demonstrated that the difference in molecular profiles between the two groups was substantially larger than the corresponding variation within each group. Indeed, proteins as well as fatty acids were found to be significantly altered when comparing the two groups (Fig. 4A,B).

In the context of this study, the observed signal for NOTCH3 was of particular interest. NOTCH3 is a signaling receptor typically expressed by endothelial cells. Recent evidence indicates that the NOTCH pathway is essential for the proper formation and regulation of the vasculature^37^. Furthermore, it plays an important role particularly in the development, function, and maintenance of vascular smooth muscle cells in vivo^38^. More specifically, NOTCH3 has been shown to be crucial for interactions of pericytes with the endothelium^37^, both of which are important players in the determination of vascular tone and as a consequence in the regulation of blood flow. The down-regulated proteins JUP and CHL1 are cell adhesion molecules typically released via exosomes. Remarkably, JUP has been demonstrated to be an integral part of insulin receptor^39^. The presently observed down-regulation of JUP after COVID infection, potentially caused by protein nitration, may thus account for a disturbance in insulin signaling and metabolic control. Insulin resistance was already identified as risk factor for the development of long COVID symptoms^40^. The two up-regulated proteins found are antibody chains, indicating a specific antibody response, as expected.

The general down-regulation of fatty acids (Fig. 4) may indicate a reduction of lipase activities. The accompanying increase of triglycerides (Fig. 4C) points to a reduction of lipoprotein lipase as already reported after COVID-19 Infection^41^. Although in the literature a positive association between BMI and postprandial triglyceride levels was reported, we deem it unlikely that elevated triglycerides in the COVID 19 group can be explained by increased BMI as this correlation was limited until 2 h of the meal^42^.

Lipoprotein lipase activity has been described to be down-regulated upon nitration^43^, a chemical reaction promoted by nitric oxide. Nitric oxide is, besides many other essential functions in different concentration ranges, a powerful antiviral agent strongly induced upon viral infections^44^. High concentrations of nitric oxide may cause protein nitration resulting in protein dysfunction, as already described in case of cell adhesion molecules subsequently downregulated in endothelial cells after infection^45^. Nitration and subsequent degradation may thus also account for the presently observed downregulation of cell adhesion molecules JUP and CHL1. Importantly, the patients recovered from a COVID-19 infection show no signs for such inflammation, as this would be easily detectable by plasma proteome profiling. In addition, in contrast to the present observations, inflammation would rather increase plasma fatty acid levels and eicosanoid levels due to the activation of phospholipase A2^46^. Phospholipase A2 preferentially releases polyunsaturated fatty acids (PUFAs) followed by their enzymatic oxidation to form eicosanoids^47^.

To sum up, viral infections are well described to induce nitric oxide during acute inflammation, which may cause lipoprotein lipase and cell adhesion molecule nitration and degradation. These events may thus well explain the present observations including the downregulated fatty acids and eicosanoids in the patients after a COVID-19 infection. However, COVID-19 infection has been well documented to be associated with persisting oxidative stress-induced endothelial dysfunction and subsequently decreased vascular nitric oxide formation^48^. It is thus consequent to assume that, after COVID-19 infections, the nitric oxide levels may go down transiently below normal levels as indicated by a recent study^49^. This lack of nitric oxide may account for the impaired microvascular perfusion described above. Thus, here we suggest a biphasic disease model assuming massive protein damage during the acute pro-inflammatory phase by high nitric oxide levels and subsequently other but still serious effects due to a lack of nitric oxide synthesis. An adaptive response to nitric oxide synthase activation, inhibition by tyrosine nitration as described above, may account for this apparent paradox and is well documented^50^.

### Strengths and limitations of the study

Our study has strengths and limitations that warrant discussion. The strength of our approach is the use of multimodal imaging for the non-invasive in-vivo assessment of ocular perfusion and oxygen metabolism in the retina, which allows us to directly draw conclusions on the microcirculation. We have recently used this approach to assess functional changes in other systemic diseases such as diabetes or multiple sclerosis^32,51^. The use of state-of-the-art molecular profiling allowed us to get more insight in the molecular mechanisms that may be responsible for the observed changes in the ocular vasculature and to suggest a new disease model. We have not yet direct evidence for the involvement of nitric oxide as suggested above. However, the present observations will guide studies specifically designed to answer these highly relevant questions.

Another limitation of the current study is the small sample size, the unequal number of subjects per group and the cross-sectional nature of the experimental design. Thus, no data of the pre-infectious period is available and, based on our results, we cannot draw conclusions regarding the time-course of the observed changes. Another issue is that BMI was significantly higher in patients that had recovered from moderate to severe COVID-19 infection compared to healthy controls. This may not have happened by chance, as it has already been well established that obesity is a risk factor for a more severe course of the disease^52–54^. A recently conducted study found a positive association between BMI and vessel density in the SVP in healthy Chinese adults^55^. In our study, no difference in vessel density in the SVP between the two groups was found although BMI was significantly higher in the COVID-19 group, as mentioned above. This could either be due to the observed retinal vasoconstriction in the COVID-19 group, counteracting on the vasodilation that would otherwise have been observed in obese patients or it could be caused by differences between the study populations. Another reason could be differences in the analysis algorithm used for determination of vessel density. Also, we only matched the groups according to age and sex, not according to BMI. A larger, longitudinal study would be necessary to overcome these limitations.

## Conclusion

In summary, we found evidence for altered retinal vessel calibers, retinal perfusion parameters and oxygen metabolism to persist in patients up to 6 months after full recovery from a COVID-19 infection. This finding is supported by molecular profiling analysis of blood plasma, demonstrating the deregulation of vascular cell adhesion molecules and indicating a down-regulation of nitric oxide-related endothelial and immunological cell functions after COVID-19 infection. Further longitudinal studies are needed to investigate whether ocular perfusion parameters are suitable biomarkers for risk assessment after a COVID-19 infection.

### Supplementary Information

Supplementary Information 1.

Supplementary Information 2.

Supplementary Information 3.

## Data Availability

The data presented in this study are available on reasonable request from the corresponding author.
