# Supplementary material for: Impaired retinal oxygen metabolism and perfusion are accompanied by plasma protein and lipid alterations in recovered COVID-19 patients
Source: Sci Rep. 2024 Apr 10;14:8395. doi: 10.1038/s41598-024-56834-4 (PMC11006918; doi:10.1038/s41598-024-56834-4)
Supplement: Supplementary file 1 — Supplementary Information 1. [file 41598_2024_56834_MOESM1_ESM.docx]

**Supplementary Information File**

**Impaired retinal oxygen metabolism and perfusion are accompanied by plasma protein and lipid alterations in recovered COVID-19 patients**

Viktoria PAI^1^, MD, Andrea BILECK, PhD^2,3^, Nikolaus HOMMER, MD^1^, Patrick JANKU, BSc^1^, Theresa LINDNER; MD^1^, Victoria KAUER, MD^1,4^, Benedikt RUMPF, MD^4^, Helmuth HASLACHER, MD, PhD^5^, Gerhard HAGN, MSc^2^, Samuel M. MEIER-MENCHES, PhD^2,3,6^, Leopold SCHMETTERER, PhD^1,7-12^, Doreen SCHMIDL, MD, PhD^1^, Christopher GERNER, PhD^2,3^, Gerhard GARHÖFER, MD^1^

1 Department of Clinical Pharmacology, Medical University of Vienna, Austria

2 Department of Analytical Chemistry, Faculty of Chemistry, University of Vienna, Austria

3 Joint Metabolome Facility, University of Vienna and Medical University Vienna, Austria

4  Department of Medicine IV for Infectious Diseases and Tropical Medicine, Clinic Favoriten, Vienna, Austria

5  Department of Laboratory Medicine, Medical University of Vienna, Austria

6 Institute of Inorganic Chemistry, Faculty of Chemistry, University of Vienna, Austria

7 Singapore Eye Research Institute, Singapore National Eye Centre, Singapore

8 Ophthalmology and Visual Sciences Academic Clinical Program, Duke-NUS Medical School, Singapore

9 SERI-NTU Advanced Ocular Engineering (STANCE), Singapore, Singapore

10 School of Chemistry, Chemical Engineering and Biotechnology, Nanyang Technological University, Singapore

11 Center for Medical Physics and Biomedical Engineering, Medical University of Vienna, Vienna, Austria

12 Institute of Molecular and Clinical Ophthalmology, Basel, Switzerland

**S-Table 1:**

| **Diagnosis** | **Number of observations** |
| --- | --- |
| Adipositas | 1 |
| Allergy | 10 |
| Angina pectoris | 1 |
| Arterial hypertension | 7 |
| Asthma bronchiale | 2 |
| Atrial fibrillation | 1 |
| Benign prostatic hyperplasia | 1 |
| Chronic kidney failure | 2 |
| Diabetes Type 2 | 3 |
| Gastroesophageal reflux disease | 1 |
| Hypercholesterolemia | 2 |
| Hyperlipidemia | 1 |
| Hypothyreosis | 1 |
| Membranous glomerulonephritis | 1 |
| Migraine | 1 |
| Peripheral arterial disease | 1 |
| Ventricular septal defect | 1 |

*Concomitant diseases of patients in the COVID-19 group.*
